# Identification of Candidate Genes Conferring Cold Tolerance to Rice (*Oryza sativa* L.) at the Bud-Bursting Stage Using Bulk Segregant Analysis Sequencing and Linkage Mapping

**DOI:** 10.3389/fpls.2021.647239

**Published:** 2021-03-11

**Authors:** Luomiao Yang, Lei Lei, Peng Li, Jingguo Wang, Chao Wang, Fan Yang, Jiahui Chen, HuaLong Liu, Hongliang Zheng, Wei Xin, Detang Zou

**Affiliations:** Key Laboratory of Germplasm Enhancement, Physiology and Ecology of Food Crops in Cold Region, Ministry of Education, Northeast Agricultural University, Harbin, China

**Keywords:** *Oryza sativa* L., cold tolerance, bulk segregant analysis sequencing, linkage mapping, bud-bursting

## Abstract

Low-temperature tolerance during the bud-bursting stage is an important characteristic of direct-seeded rice. The identification of cold-tolerance quantitative trait loci (QTL) in species that can stably tolerate cold environments is crucial for the molecular breeding of rice with such traits. In our study, high-throughput QTL-sequencing analyses were performed in a 460-individual F_2__:__3_ mapping population to identify the major QTL genomic regions governing cold tolerance at the bud-bursting (CTBB) stage in rice. A novel major QTL, *qCTBB9*, which controls seed survival rate (SR) under low-temperature conditions of 5°C/9 days, was mapped on the 5.40-Mb interval on chromosome 9. Twenty-six non-synonymous single-nucleotide polymorphism (nSNP) markers were designed for the *qCTBB9* region based on re-sequencing data and local QTL mapping conducted using traditional linkage analysis. We mapped *qCTBB9* to a 483.87-kb region containing 58 annotated genes, among which six predicted genes contained nine nSNP loci. Quantitative reverse transcription-polymerase chain reaction (qRT-PCR) analysis revealed that only Os09g0444200 was strongly induced by cold stress. Haplotype analysis further confirmed that the SNP 1,654,225 bp in the Os09g0444200 coding region plays a key role in regulating the cold tolerance of rice. These results suggest that Os09g0444200 is a potential candidate for *qCTBB9*. Our results are of great significance to explore the genetic mechanism of rice CTBB and to improve the cold tolerance of rice varieties by marker-assisted selection.

## Introduction

Rice (*Oryza sativa* L.) is an important food crop that has adapted to the tropical climate of the world. However, chilling injury of rice that occurs at high latitudes and high altitudes has severely restricted its production (Jena et al., 2012; Liu C. T. et al., 2019). Fast and uniform seed germination and vigorous seedling growth are essential for stable crop growth. The germination rate and early growth of seedlings after germination are the two main characteristics that are directly related to seedling vigor. Cold stress usually damages and delays germination and seedling growth of rice, leading to inferior stand establishment and uneven maturity, especially in areas where direct-seeded rice and cultivation are performed under low local temperatures (Iwata and Fujino, 2010; Manangkil et al., 2013). Therefore, low-temperature sensitivity at the bud-bursting stage remains a major challenge for direct-seeded rice. Improved cold tolerance at the bud-bursting (CTBB) stage is an important agronomic characteristic of direct-seeded rice breeding programs.

Because of the complexity and polygenicity of cold tolerance, numerous efforts have been directed to the detection and mapping of quantitative trait loci (QTL). In the past 20 years, more than 550 QTLs have been reported for different growth stages (germination, seedling, and reproductive/booting stage) for low-temperature stress using molecular markers of different genetic backgrounds derived from bi-parental mapping populations and diverse genetic resources of rice accessions (Najeeb et al., 2020). For example, numerous double haploid lines, backcross-inbred lines, recombinant inbred lines (RIL), and near-isogenic lines have been developed, with cold-tolerant varieties such as Kunming Xiaobaigu (Dai et al., 2004), Lijiang Xintuanheigu (Shirasawa et al., 2012), Koshihikari (Endo et al., 2016), M202 (Andaya and Tai, 2006), Norin-PL8 (Saito et al., 2010), and a Chinese type of wild rice (Dongxiang wild rice) (Mao et al., 2015) as donors. These representative cold-tolerant germplasms have contributed valuable QTLs or genes for research on the cold tolerance of rice. However, fewer QTLs have been identified at the bud-bursting stage than at the other developmental stage. As of 2015, 29 QTLs related to CTBB were integrated into chromosomes through meta-analysis (Zhu et al., 2015). Among them, *qCTBP2, qCTBP4*, and *qCTBP7* were mapped using F_3_ populations based on simple sequence repeat (SSR) markers (Qiao et al., 2005). Four QTLs on chromosomes 3, 7, and 12 were detected using 98 backcross-inbred lines (Yang et al., 2009). In total, four QTLs for CTBB were preliminarily mapped on chromosomes 5 and 7 using a set of 95 chromosome segment substitution lines (CSSLs) derived from indica rice 9311 and Japonica rice Nipponbare (Lin et al., 2010). In the past 5 years, five new QTLs have explained 4.17–6.42% of the total phenotypic variation explained during the bud-bursting stage based on an RIL derived from H335 and CHA-1 (Yang et al., 2020). The other two cold-tolerant QTLs (*qCTBB-5* and *qCTBB-6*) at the bud-bursting stage were mapped using single-segment substitution lines (Yang et al., 2016). These results indicate that the research on CTBB of rice is still in its infancy. Most QTLs lie within a range of 10–30 cM through primary populations, and it is difficult to perform molecular marker-assisted breeding. To date, only two QTLs have been cloned; *qLTG3-1* encodes a protein with unknown function, which might regulate the vacuolization of aleurone and epidermal cells, thereby causing relaxation of these tissues and enhancing the germination potential of seeds at low temperatures (Fujino et al., 2008; Fujino and Matsuda, 2010; Fujino and Sekiguchi, 2011), whereas *OsSAP16* encodes a zinc-finger domain protein and serves as the major causal gene for low-temperature germinability (Wang et al., 2018). These examples demonstrate that the molecular regulation of CTBB is extremely complex, and the aforementioned QTLs were identified by bi-parental cross-linkage mapping, which is a labor- and time-intensive method to map genotypes of a large number of individuals in a segregated population. Therefore, to obtain reliable QTLs, it is necessary to implement a fast and efficient method to identify QTLs.

Recently, with the rapid development of next-generation sequencing (NGS) technology, the combination of bulked segregant analysis with NGS has become an important path to mine QTLs and genes (Abe et al., 2012; Vikram et al., 2012; Kadambari et al., 2018; Wambugu et al., 2018). Compared with traditional linkage mapping, QTL-sequencing (Seq) can improve work efficiency and provide high-density variation. The efficiency of QTL-Seq has been successfully demonstrated in many plants, such as cucumber (Lu et al., 2014), soybean (Song et al., 2017), rice (Wambugu et al., 2018), and tomato (Wen et al., 2019). Although the technique allows for the quick identification of major QTLs, confidence interval (CI) resolution is still rough. Therefore, researchers have to combine QTL-Seq with fine-mapping (Zhang X. et al., 2018) and RNA-Seq (Park et al., 2019) to identify candidate QTL genes. For example, *CaqSW1.1* (Das et al., 2015) was mapped from 1.37 Mb to 35 kb by QTL-Seq and classical QTL mapping, and the final candidate genes were identified. In another case, four candidate genes (*SlCathB2, SlGST, SlUBC5*, and *SlARG1*) associated with heat tolerance were detected by QTL-Seq and RNA-Seq in tomato (Wen et al., 2019). Thus, the method of obtaining a major QTL interval by QTL-Seq combined with fine-mapping to mine target genes provides a new perspective.

In our study, two strategies, QTL-Seq followed by linkage mapping, were employed to identify the major QTL interval for CTBB in rice. An F_2__:__3_ population derived from a cross between Dongnong430 (DN430, cold-sensitive variety) and Dongfu104 (DF104, strongly cold-tolerant variety) was used for QTL-Seq analysis. Furthermore, a linkage mapping strategy was considered to anchor major QTLs using a KASP marker. Furthermore, quantitative reverse transcription-polymerase chain reaction (qRT-PCR) and haplotype analysis were employed to verify the association between candidate genes and cold tolerance. The results obtained in this study are expected to help reveal the mechanism by which cold sensitivity changes into cold tolerance in rice at the molecular level and will improve the understanding of mechanisms underlying CTBB in rice.

## Materials and Methods

### Plant Materials

In this study, two Japonica varieties, the cold-sensitive female parent Dongnong 430 (DN430) and the cold-tolerant male parent Dongfu104 (DF104), were used as the parental lines to develop the 460 F_2__:__3_ lines. The materials mentioned previously herein were all obtained from Northeast Agriculture University in the northeast region of China.

### Cold-Tolerance Evaluation at Bud-Bursting Stage

One hundred seeds of each F_2__:__3_ family and two parental were incubated at 40°C for ∼36 h to break dormancy and were soaked in deionized water at 30°C for approximately 72 h for germination. Fifty 5-mm-long buds of the two parental seeds were selected, washed with sterile water, and transferred to a petri dish with two layers of filter paper. In addition, 50 germinated seeds from 460 progeny lines were transferred to 96-well PCR plates. These 50 germinated seeds were then further subjected to a cold treatment at 5°C for 9 days in a growth chamber in darkness and were allowed to resume growth at 28°C for 3 days to investigate the severity of damage (SD). The SD of all 460 F2:3 lines and their parents was evaluated using the following scale: a cold score of 0 indicated that the seedling had normal leaf color and grew well with no damage, to a cold score of 7, which indicated that the germinated seed was dead with no green leaves, as described in previous studies (Zhang M. et al., 2018). The SD value of a plant represented the average score of 50 seeds. The seed survival rate (SR) was evaluated after 7 days of recovery of growth and was calculated as follows: SR (%) = (surviving seedlings/50) × 100 (Zhou et al., 2012). Three biological replicates were performed.

### Construction of Segregating Pools

Young leaves of the 460 F_2_ individuals were collected separately for total genomic DNA extraction using a minor modified cetyltrimethylammonium bromide (CTAB) method (Murray and Thompson, 1980). The genomic DNAs of 30 extremely cold-tolerant and 30 extremely cold-sensitive individuals were selected as two bulked pools according to the SR of the F_2__:__3_ population, ranging from 0 to 100%. To simplify the following description, we abbreviate DF104 as T, DN430 as S, the cold-tolerant pool as the T-pool, and the cold-sensitive pool as the S-pool. Isolated DNA was quantified using a Nanodrop 2000 spectrophotometer (Thermo Scientific, Fremont, CA, United States). All DNAs from the T-pool and S-pool plants were quantified on a Qubit^®^ 2.0 Fluorometer (Life Technologies, Carlsbad, CA, United States), and equal amounts of DNA from the T-pool and S-pool plants were mixed.

### NGS Sequencing and QTL-Seq Analysis

Total genomic DNA was extracted from bulked pools, and at least 3 μg genomic DNA was used to construct paired-end libraries with an insert size of 500 bp using the Paired-End DNA Sample Prep kit (Illumina Inc., San Diego, CA, United States). These libraries were sequenced using the HiSeq X10 (Illumina Inc., San Diego, CA, United States) NGS platform at Genedenovo (Guangzhou, China). Quality trimming is an essential step in generating high confidence in variant calling; raw reads were processed to obtain high-quality clean reads according to three stringent filtering standards as follows: (1) reads with ≥ 10% unidentified nucleotides (N) were removed; (2) reads with > 50% bases with a phred quality score of ≤ 20 were removed; and (3) reads were aligned to the barcode adapter.

To identify single-nucleotide polymorphisms (SNPs) and InDels, filtered reads were aligned to the Nipponbare reference genome sequence (International, 2005) using Burrows-Wheeler Aligner (BWA, v 0.7.16a-r1181) with parameter “mem -M”; -M is an option used to mark shorter split alignment hits as secondary alignments (Kumar et al., 2019). Variant calling was performed using the GATK UnifiedGenotyper (v3.5). SNPs and InDels were filtered using the GATK VariantFiltration function with proper standards (-Window 4, -filter “QD < 4.0 | | FS > 60.0 | | MQ < 40.0,” -G_filter “GQ < 20”). All mutations were annotated for genes and functions, as well as genomic regions, using ANNOVAR (Wang et al., 2010). Association analysis was performed using the SNP-index (Abe et al., 2012), Δ(SNP-Index) (Takagi et al., 2013), calculation of G statistic (Magwene et al., 2011; Mansfeld and Grumet, 2018), Euclidean distance (ED) (Hill et al., 2013), and two-tailed Fisher exact test (Fisher, 1922) based on the SNPs. Finally, the overlapping interval of the four methods was considered the final QTL interval.

### Development of KASP Markers and Linkage Mapping

To develop markers for validation of QTL-Seq results and narrow the candidate region, 26 non-synonymous SNPs (nSNPs) with significant peaks were found based on the CDS sequence of the two parents in the *qCSBB9* region under the four association analysis algorithms [Δ (SNP-Index), G-value, ED-value, and Fisher exact test] and were considered as further markers for linkage mapping. KASP primers were designed using Primer express 3.0.1^1^; the primers are listed in Supplementary Table 1. All markers were selected for polymorphism between parents, and genotyping of the 460 individuals was performed using the polymorphic markers, which were used to construct the linkage map and narrow the candidate region using the inclusive composite interval mapping (ICIM) module of QTL IciMapping 4.2^2^ The threshold of the logarithm of odds (LOD) for declaring the presence of a significant QTL was determined by the permutation test with 1,000 repetitions at *P* < 0.01.

### Prediction of Candidate Genes for the CTBB

To predict possible candidate genes for the CTBB, the following three strategies were comprehensively considered in this study. First, candidate gene prediction was performed to compare the DNA sequences of those genes within the QTL regions using the whole-genome DNA resequencing database of the parents. In the QTL regions, we focused on open reading frames with non-synonymous mutant SNPs between the two parents. For the second, the most possible candidate genes were reselected according to their functional annotations, screening the rice genome database^3^. For the third, gene functions were annotated by gene ontology (GO) (Ashburner et al., 2000) and Kyoto Encyclopedia of Genes and Genomes (KEGG) (Kanehisa et al., 2004) databases with BLAST software (Altschul et al., 1997). Finally, the candidate genes were obtained.

### Verification of the Expression Level of Candidate Genes

Five-millimeter-long buds of DN430 and DF104 were collected at 2, 6, 12, 24, and 48 h after cold treatment for three repetitions, frozen in liquid nitrogen, and stored at −80°C for total RNA extraction. Control plants were also collected and stored similarly. qRT-PCR was used to quantify the expression levels of candidate genes under cold treatment. Total RNA was extracted from rice tissues using TRIzol reagent (Thermo Fisher Scientific, Waltham, MA, United States) and treated with DNase I to eliminate any DNA contamination. RNA quality was assessed by electrophoresis, and the RNA was stored at −80°C until use. First-strand cDNA (10 μL) was synthesized according to the instructions for the PrimeScript^TM^ RT Master Mix [Takara Biomedical Technology (Beijing) Co., Ltd., Beijing, China]. Primers were designed with Primer Premier v. 5.0 (PREMIER Biosoft International, United States) and are listed in Supplementary Table 2. The housekeeping gene *Actin1* (Os05g36290) was used as an internal control (Siahpoosh et al., 2012), and qRT-PCR was performed using a Roche LightCycler 2.10 with a 2× SYBR Green I PCR Master Mix. qRT-PCR analysis was performed as previously described (Zhang Z. et al., 2018).

### Haplotype Analysis of Candidate Genes

Here, we present one method of analyzing haplotypes of candidate genes, which are variants of candidate genes in the 30 T-pool, 30 S-pool, and 295 northern China Japonica rice strains (Li et al., 2019). Primer sequences were designed for candidate genes at SNP sites between parents, and the DNA fragments of the sequences in 60 lines were amplified by PCR to perform haplotype statistics on the distribution of target genes. All specific primer sequences are listed in Supplementary Table 2. The PCR reaction mixture had a total volume of 20 μL, containing 1.5 μL of forward primer (10 μm), 1.5 μL of reverse primer (10 μm), 2 μL of genomic DNA (50 ng/μL), 5 μL of ddH_2_O, and 10 μL of Pfu master mix (Chinese Beijing cwbio), which included Taq DNA polymerase, PCR buffer, Mg^2+^, and dNTPs. The PCR reaction was performed in an Eppendorf 5333 Mastercycler using the same protocol as that used for qRT-PCR (Zhang Z. et al., 2018). The products were examined by 1% agarose gel electrophoresis. Direct sequencing of PCR products was performed by BGI Life Technology Co., Ltd.

## Results

### Phenotypic Analysis of the Parental Lines and F_2__:__3_ Population

**FIGURE 1:**
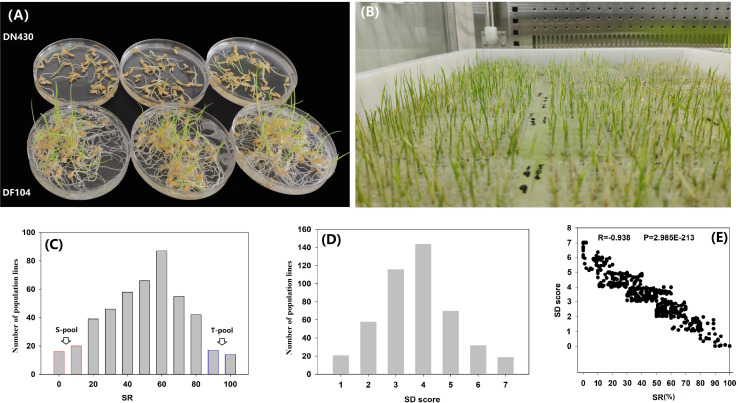
Phenotypic analysis of cold tolerance in rice bud-bursting stage. **(A)** The performance of DN420 and DF104 under cold treatment (5°C/9 days). **(B)** Identification of cold tolerance of F_2_ population in artificial climate chamber. **(C,D)** Frequency distribution of seed survival rate (SR) and severity of damage (SD) variation measured among 460 mapping individuals of F_2__:__3_ populations. **(E)** Paired scatter plot analysis of SR and SD. T-Pool, DNA pool of cold-tolerant lines, S-pool, and DNA pool of cold-sensitive lines.

Two parental varieties, DN430 and DF104 (Figure 1A), along with their 460 F_2__:__3_ lines (Figure 1B), were evaluated for two cold tolerance indices (SD and SR) at the bud-bursting stage at 5°C/9 days. The SR of the cold-tolerant variety DF104 was significantly higher than that of cold-sensitive DN430, indicating that DF104 had a stronger CT than DN430. In the F_2__:__3_ population, SR exhibited continuous variation from 0 to 100% (Figure 1C and Supplementary Table 3), and SD showed variation from 0.00 to 7.00 (Figure 1D and Supplementary Table 3). Interestingly, the scatter plot revealed that all individuals with SD values of 0 or 1 had SR values between 90 and 100%, whereas lines with SD values of 5 or 7 had SR values distributed between 0 and 20% (Figure 1E). Correlation analysis showed that the correlation coefficient between SR and SD was −0.938 (*P* = 2.985E-213). Figure 1C shows that among the 460 lines, 30 cold-tolerant and 30 cold-sensitive lines were identified. These were then selected to prepare the T-pool and S-pool, respectively, which were then used for DNA re-sequencing.

### Whole-Genome Re-sequencing and QTL-Seq Analysis

Illumina high-throughput sequencing generated 566.87 million raw reads; 562.53 million clean reads (99.20%) were obtained after filtering, and the four samples generated 84,033,193,174-bp bases. When compared with the “Nipponbare” reference genome, these four samples generated 23,53,180 SNPs and 4,16,850 Indels (Supplementary Table 4). These high-quality data provided a solid foundation for subsequent analysis.

**FIGURE 2:**
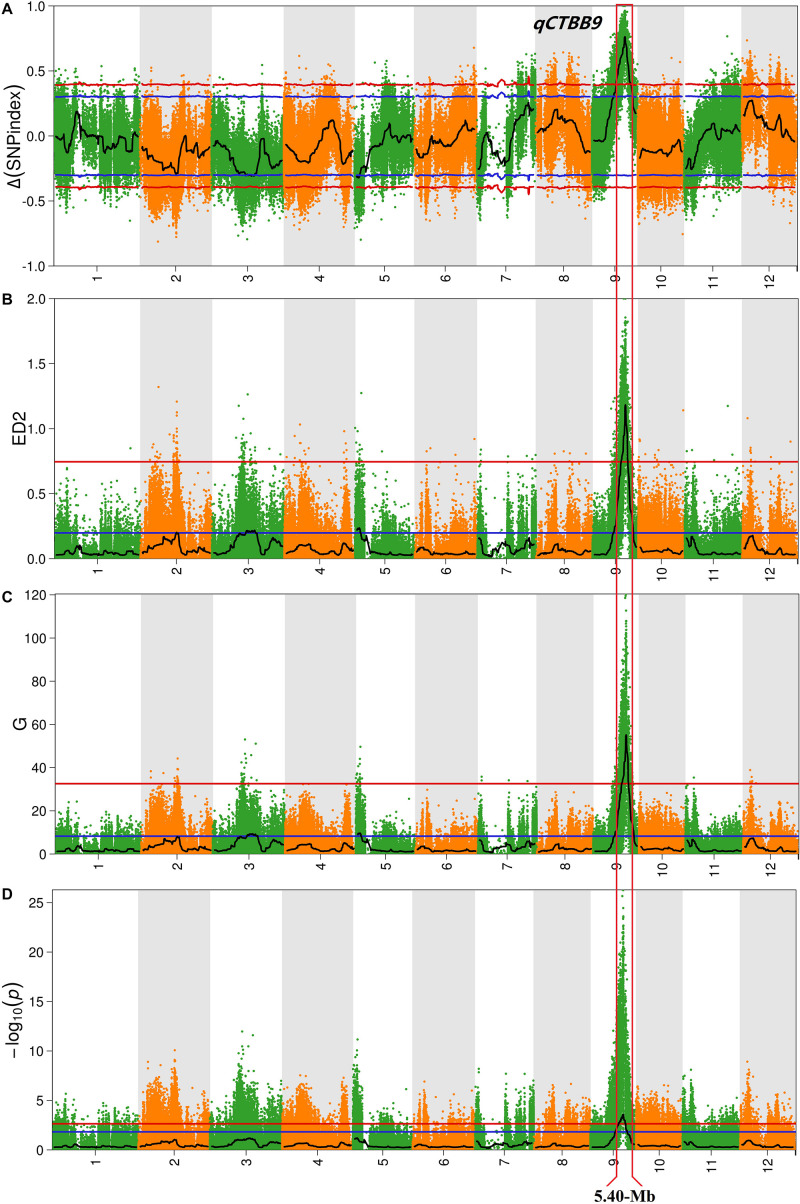
Analysis of quantitative trait loci of cold tolerance at the bud-bursting stage based on QTL-Seq. **(A)** Manhattan plot shows the distribution of SNP-index and Δ (SNP-index) on chromosomes. **(B)** The Manhattan plot shows the distribution of the square of the Euclidean distance on the chromosomes. **(C)** The Manhattan plot shows the distribution of G′-Value on the chromosomes. **(D)** The Manhattan plot shows the distribution of –log_10_(P) value on the chromosomes based on Fisher exact test. The blue and red lines represent 95 and 99% confidence intervals, respectively.

For all obtained SNPs, using the association threshold of 95 and 99%, the values of Δ (SNP-Index) (Figure 2A), ED (Figure 2B), G′ value (Figure 2C), and Fisher’s exact test (Figure 2D) were used to intercept the association interval for CTBB. There were four QTL regions to be identified, which mapped to chromosome 2, 3, 5, and 9 (Table 1 and Figure 2). For chromosome 2, one genomic region (17.06–19.24 Mb) was obtained by the ED algorithm at the 95% significance level, which we designated *qCTBB2*. The other three genomic regions, namely, *qCTBB3* (16.32–22.58 Mb), *qCTBB5* (0–3.54 Mb), and *qCTBB9* (9.54–21.38 Mb) on chromosomes 3, 5, and 9 for CTBB at the 95% significance level were revealed. However, the highest peak-value could be seen on chromosome 9 in the four algorithms. Of note, only a single QTL, *qCTBB9*, was obtained when the cut-off CI value of 99% was applied. For Δ (SNP-Index), *qCTBB9* spanned 9.34 Mb intervals, and the ED algorithm completely included the result given by the Δ (SNP-Index) algorithm. The G′ value and Fisher’s exact test similarly captured the results of the first two algorithms. Here, the ED, G-value, and Fisher’s exact test identified a small QTL interval. Obviously, a larger peak-value was obtained at the 99% significance level than at the 95% significance level. Therefore, *qCTBB9* is considered the most significant target for CTBB; considering the overlap interval of the four calculation models, *qCTBB9* was narrowed to a region of 5.4 Mb, which contains 647 predicted genes. According to QTL-Seq analysis, for the *qCTBB9* coding region, 281 nSNPs and seven Indels were detected (Supplementary Table 5).

**TABLE 1:** QTLs conferring cold tolerance in four method identified using QTL-seq.

**QTL names**	**Calculation models**	**CI**	**Chr**	**Start (bp)**	**End (bp)**	**Peak**	**Overlapping region (Mb)**
*qCTBB2*	Euclidean distance	0.95	2	17,060,001	19,240,000	0.201677	17.06–19.24
*qCTBB3*	Δ(SNP-index)		3	16,320,001	22,580,000	–0.30825	16.32–22.58
	G′ value			16,140,001	23,600,000	9.325734	
	Euclidean distance			16,020,001	22,980,000	0.217969	
*qCTBB5*	Δ(SNP-index)		5	1	3,540,000	–0.3219	0–3.54
	G-statistic			1	3,660,000	9.641273	
	Euclidean distance			1	3,800,000	0.235656	
*qCTBB9*	Δ(SNP-index)		9	9,960,001	21,380,000	0.76226	9.54–21.38
	G′ value			9,580,001	21,500,000	55.11879	
	Euclidean distance			9,540,001	21,540,000	1.182111	
	Fisher’s exact test			11,540,001	19,560,000	0.001811	
	Δ(SNP-index)	0.99	9	11,160,001	20,500,000	0.76226	13.38–18.74
	G-statistic			13,340,001	18,740,000	55.11879	
	Euclidean distance			13,380,001	18,740,000	1.182111	
	Fisher’s exact test			13,200,001	18,740,000	0.001811	

### Fine Mapping of *qCTBB9*

**FIGURE 3:**
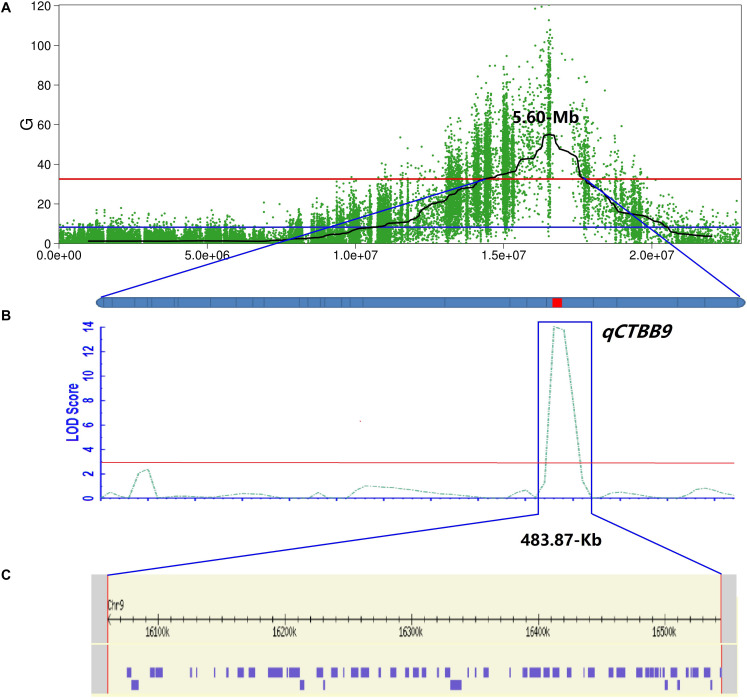
Further mapping of the *qCTBB9*. **(A)** The Manhattan diagram shows the location of the *qCTBB9* on chromosome 9. **(B)** *qCTBB9* detected by using the ICIM module of QTL IciMapping 4.2. Linkage map based on KASP markers. The red lines represent the logarithm of odds (LOD) threshold of 2.0. **(C)** 58 genes in *qCTBB9* region were obtained through the annotation information on the *Nipponbare* genome.

Because the 5.4 Mb region still contained numerous genes, to fine-map *qCTBB9* based on the nucleotide polymorphism information of the 5.4 Mb it covers, the highly credible SNPs obtained by the four association algorithms were fully considered, and the SNPs with low credibility under some algorithms were filtered out. Finally, SNPs with a relative distance of less than 200 bp were eliminated, and 26 excellent nSNPs (Supplementary Table 1) were used to scan the genotypes of the 460 lines and were used to obtain a *qCTBB9* linkage map. A QTL was considered significant when the LOD value was equal to or larger than 3.0 (Zhao et al., 2018). The SR of the 460 lines under cold stress was analyzed by linkage mapping. *qCTBB9* was simultaneously linked with SR and anchored to the 483.87 kb interval between 1,60,59,891 and 1,65,43,763 bp (Figures 3A,B); this explained 24.91% of the phenotypic variation in seed SR, and the peak-value for the LOD score was 14.01 (Table 2). The positive allele *qCTBB9* was contributed by DF104, and *qCTBB9* was optimized to a physical interval of 483.87 kb.

**TABLE 2:** Identification of *qCTBB9* for cold tolerance at bud-bursting stage by linkage analysis.

**Trait**	**Position (cM)**	**LeftMarker**	**RightMarker**	**LOD**	**PVE(%)**	**Add**	**LeftCI**	**RightCI**
SR	48	C9_16059891	C9_16543763	14.01	24.91	−4.09	47.5	49.5

### Putative Candidate Genes and Candidate SNPs in the Genomic Regions for CTBB

The 483.87 kb interval was intercepted through the annotation information of the Nipponbare genome^3^, and 58 annotation genes were captured (Supplementary Table 6 and Figure 3C). Some important genes or gene families related to cold tolerance in rice or other crops have been reported, such as cytochrome P450 (Su et al., 2010), a hypothetical protein (Zhao et al., 2017), a chromatin remodeling factor (Yang et al., 2019), an F-box domain (Saito et al., 2010), lipid metabolism genes (Zhang et al., 2019), a protein kinase (Ding et al., 2019), and a TATA-binding protein (Xu et al., 2020). Although similar genes or proteins were included in the list of 58 candidate genes, the 43 genes obtained from GO analysis were not annotated to the term response to cold. The most enriched terms of biological process, molecular function, and cellular component ontology were metabolic processes (i.e., GO:0006629, GO:0008152, and GO:0044237), activity (i.e., GO:0016491, GO:0003824, and GO:0016787), and cell (i.e., GO:0005575, GO:0005623, and GO:0044464), respectively (Supplementary Table 7 and Supplementary Figure 1). Further, 483.87 kb on chromosome 9 had 240 effective SNPs between parental lines and a Δ (SNP−index), G-value, ED, and *P*-value of the two-tailed Fisher exact test higher than the statistical confidence at *P* < 0.01 (Supplementary Table 6). Of the 240 SNPs, 206 SNPs were intergenic and 34 SNPs were genic, including 16 intronic, nine non−synonymous, four synonymous, and five in UTRs. The nine nSNPs could be identified using both parents as a reference and affected six candidate genes encoding three hypothetical conserved genes (Os09g0436500, Os09g0443400, and Os09g0443700), one TATA-binding related factor domain containing protein (Os09g0443500), one similar to minus dominance protein (Os09g0444100), and one cholesterol acyltransferase family protein (Os09g0444200) (Table 3). As mentioned, these six types of genes might play an important role in responding to cold stress in plants. Of the six candidate genes, only Os09g0444200 was screened in the KEGG pathway (pathway ID K00679) (Supplementary Table 7 and Supplementary Figure 2), which was involved in the glycerolipid metabolism, and glycerolipid is considered significantly related to plant cold sensitivity (Eriksson et al., 2011). Based on this, we identified the six final possible candidate genes through three approaches as follows: the possible relationship between gene response and cold stress, non-synonymous mutations in the gene coding region, and the participation of genes in specific metabolic pathways that affect cold tolerance.

**TABLE 3:** Identification of SNPs in putative candidate genes for CTBB.

**Gene ID**	**Position**	**DF-104 base**	**DN-430 base**	**T-pool base**	**S-pool base**	**Δ (SNP-index)**	**G-Value**	**ED**	**Fisher exact test**	**Structure_type**	**Function**
Os09g0436500	16059891	A	C	A	C	0.92	118.55	1.68	2.21E-26	Exonic	Hypothetical conserved gene
Os09g0443400	16501770	A	G	A	G	0.30	6.07	0.18	1.56E-02	Exonic	Hypothetical conserved gene
	16501777	T	C	T	C	0.30	6.15	0.18	2.02E-02	Exonic	
	16501891	A	G	A	G	0.48	16.64	0.46	8.02E-05	Exonic	
Os09g0443500	16508612	G	T	G	T	0.89	57.80	1.60	2.84E-13	Exonic	TATA-binding related factor domain containing protein
Os09g0443700	16517219	G	C	G	C	0.86	86.30	1.46	1.36E-19	Exonic	Hypothetical conserved gene
Os09g0444100	16533992	T	C	T	C	0.67	41.01	0.89	6.13E-10	Exonic	Similar to minus dominance protein
	16535714	G	A	G	A	0.78	82.60	1.23	4.59E-19	Exonic	
Os09g0444200	16543763	A	G	A	G	0.86	91.09	1.48	1.14E-20	Exonic	Cholesterol acyltransferase family protein

### Candidate Genes Verified by qRT-PCR and Haplotype Analysis

**FIGURE 4:**
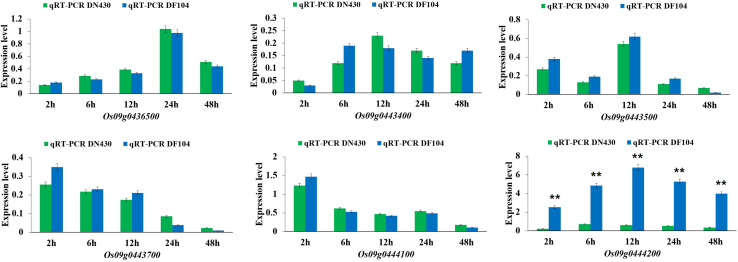
Expression levels of the six candidate genes in DN430 and DF104 after cold stress measured by qRT-PCR. The results were statistically analyzed using Student’s *t*-test (**P < 0.01).

**FIGURE 5:**
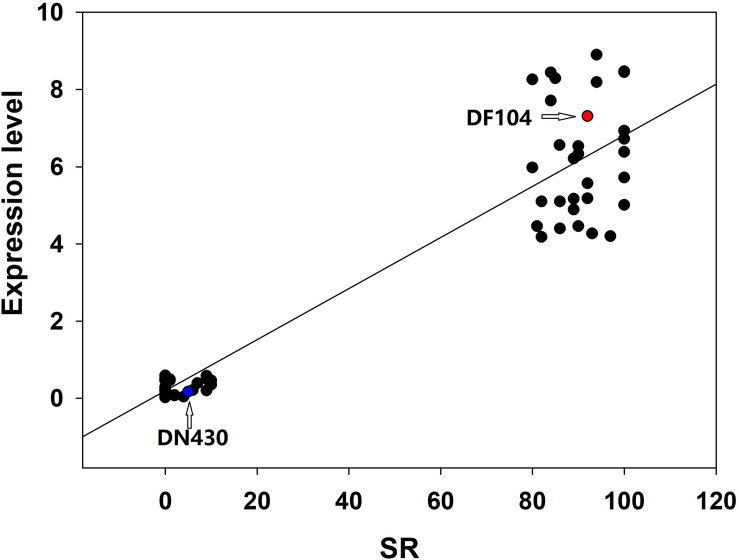
Correlation between the expression level of *Os09g0444200* and seed survival rate in 30 cold-tolerant lines and 30 cold-sensitive lines after cold treatment.

To identify strong candidate genes for *qCTBB9*, the expression levels of six genes under cold stress were further analyzed. The results showed that only Os09g0444200 showed strong induction of the cold stress response in parents with extremely strong CTBB (Figure 4), suggesting that Os09g0444200 is the most likely candidate gene for *qCTBB9*. In contrast, under normal conditions, all six candidate genes had no significant expression levels (Supplementary Figure 3). The strong correlation between RNA expression levels and SR in these 60 lines indicates the regulatory function of Os09g0444200 for CTBB (Figure 5). Based on these findings, we considered Os09g0444200 as a possible candidate CTBB gene based on *qCTBB9* for further analysis.

Os09g0444200 encodes lecithin–cholesterol acyltransferase. To reveal the significance of variations in the Os09g0444200 coding region, we selected 60 lines each from the T-pool and the S-pool and sequenced their Os09g0444200 genes by Sanger sequencing. Four SNPs were observed in the CDS sequence of Os09g0444200–SNP-16543710, SNP-16543763, SNP-16544225, and SNP-16546293. The four SNPs divided 460 F_2__:__3_ genotypes into four haplotypes (HapI, *Hap*II, HapIII, and HapIV) (Supplementary Table 8). DN430 was classified as HapIII, and DF104 was classified as HapI. The 30 cold-tolerant and 30 cold-sensitive lines showed consistent isolation of the nSNP (1,654,225 bp), suggesting that this might lead to phenotypic differences in CTBB. We then referred to the data of the 3010 Rice Genomes Project and found that HapI (TAGT) and HapIII (CGAC) mainly exist in the genotype of Japonica rice (Supplementary Table 9). We subsequently analyzed the polymorphism types of Os09g0444200 in 295 China northern Japonica rice and found that HapI did not occur in the samples (Supplementary Table 10). It could thus be inferred that HapI is a rare natural variation. Therefore, it can be used as a special functional variation in DF104 to improve the CTBB of rice varieties. However, it cannot be ruled out that other Japonica Os09g0444200 markers might have similar SNPs.

## Discussion

Chilling injury events have been frequently observed in Northeast China in the past few years (Ma et al., 2017; Shen et al., 2019). The pre-germinated rice seeds directly planted in the soil are affected by the low temperature of air and/or irrigation water, which considerably reduces the germination rate and can lead to seed death (Fujino and Matsuda, 2010). Consequently, cold-tolerant rice varieties will contribute to food security and sustainable development. To locate the major QTLs of CTBB, in this study, we used several approaches to obtain the most reliable QTL interval. First, we used a large population to identify their phenotypes in low-temperature conditions, thereby obtaining accurate cold-tolerant phenotypes for each line. Second, two DNA pools with strict cold tolerance phenotypic differences were used to perform QTL-Seq analysis. Moreover, we used four bioinformatic analysis approaches to map QTL regions at the 99% significance level. Finally, only one highly significant peak (*qCTBB9*) was detected on chromosome 9 (Table 1 and Figure 2), with the QTL mapped between 13.34 and 18.74 Mb. Some QTLs and genes for cold tolerance on chromosomes 9 have been reported in recent years. The QTL *qPGCG9-1* (Schlappi et al., 2017) was studied for the CTBB of rice, and according to the “Nipponbare” reference genome, was mapped to chromosome 9, at a position 6.83 Mb away from *qCTBB9*. *qLTS9* and *qCTS9-5* were associated with cold tolerance at the seedling stage (Lv et al., 2016), and they were located in the 22.701–22.887 and 4.304–4.488 Mb intervals on chromosome 9, respectively; their physical distances from the *qCTBB9* were 4.00 and 5.852 Mb, respectively. The location of *qCTBB9* in our study completely overlaps the previously reported region of *qCTS-9* (Zhao et al., 2017), which has been linked to cold tolerance at the seeding stage. Moreover, in the *qCTBB9* interval, *OsWRKY76* (Yokotani et al., 2013), *OsDREB1A*, and *OsDREB1B* (Ito et al., 2006) were involved in the regulatory mechanism of the rice cold-tolerance response. In addition, we did not find any cold-tolerance QTLs or genes related to the bud-bursting stage on chromosome 9. Notably, *qCTBB9* was associated with the only strong peak under the four calculation models (Figure 2). This shows that there is a significant difference in the allele ratio between the two mixed pools. Therefore, *qCTBB9* can be considered the most significant target for cold tolerance to explore candidate genes. Moreover, the other two QTL intervals, *qCTBB3* and *qCTBB5*, were detected by three approaches [Δ(SNP-index), G′ value, and ED] at the 95% significance level (Table 1); for *qCTBB3*, no existing cold-tolerance gene or QTL was found in its genome region, and in the *qCTBB5* interval, only *OsLti6b*, encoding a hydrophobic protein homologous, increased cold tolerance by overexpression (Kim et al., 2007). However, the peak of these two QTLs was less than one-third that of *qCTBB9* (Table 1 and Figure 2). As suggested by previous studies, the causative genes underlying QTL probably reside in genomic regions with the highest peak-values (Win et al., 2017). Therefore, to identify genomic regions controlling CTBB with higher probability, we applied the *P* < 0.01 cutoff rather than *P* < 0.05.

In our study, several studies confirmed that combining QTL-Seq and traditional linkage mapping to uncover a very narrow candidate region is well suited for faster gene targeting (Feng et al., 2019; Liu G. et al., 2019). To screen potential candidate genes of the *qCTBB9* interval, based on the QTL-seq results, 26 nSNP markers were further used to perform genotype scanning on all 460 lines; using this approach, we reduced the number of candidate genes within the *qCTBB9* interval defined by QTL-Seq from 647 to 58 genes. Among these 58 genes, only six had nine functional base variations. Furthermore, the expression level of Os09g0444200 in the two parents showed that it was strongly induced after cold treatment. The expression analysis of Os09g0444200 in 60 lines with differences in extreme cold responses revealed a correlation between cold-tolerance and gene expression levels (Figure 5). Notably, there were four SNP differences between the two parents in the coding region of the Os09g0444200 gene, which directly led to the difference in the expression level of the Os09g0444200 gene under cold stress (Figure 4). Further haplotype analysis results show that the nSNP (1,654,225 bp) is responsible for the phenotypic difference (Supplementary Table 8). In addition, the 3010 Rice Genome Project database showed that the Os09g0444200^DF104^ and Os09g0444200^DN430^ haplotypes mainly exist in the genotype of Japonica rice (Supplementary Table 9). However, haplotype events did not occur in the 295 northern cultivars (Supplementary Table 10). This means that the four SNPs in the DF104 coding region are rare variants of Japonica rice in northern China, which could serve as a genomic marker for improved CTBB. Moreover, the molecular mechanism of the interaction between Os09g0444200 and its cofactors needs to be investigated in future studies. Although we have found correlations between the nSNP in Os09g0444200 and the studied phenotypic traits, this is not enough to assign a certain tolerant phenotype to a single SNP. In contrast, we believe that a favorable allelic variant in one key gene is not sufficient to provide cold tolerance, and the final cold-tolerance phenotype of the plant should be considered a result of a combination of favorable allelic variations from different key genes.

Cold tolerance is a complex trait controlled by multiple genes (Zhang et al., 2014), and the genes that have been isolated thus far can be divided into two different types, those that regulate gene expression during stress responses, including signaling components and transcription factors (TFs) (Huang et al., 2012), and those that have specific functions and participate in systemic metabolism to defend against cold stress (Huang et al., 2012). For example, *OsLTPL159* enhances the cold tolerance of rice at the early seedling stage by decreasing the toxic effect of reactive oxygen species, enhancing cellulose deposition in the cell wall, and promoting osmolyte accumulation (Zhao et al., 2020). Moreover, *CTB4a*, as a leucine-rich repeat receptor-like kinase, positively regulates the activity and content of ATP under cold stress by interacting with the β subunit of ATP synthase, thereby increasing pollen fertility (Zhang et al., 2017). In our study, we observed that Os09g0444200 encodes a lecithin–cholesterol acyltransferase through the annotation information of the Nipponbare genome, and KEGG database annotation results revealed that Os09g0444200 is involved in the lipid metabolism pathway (ko01100//Metabolic pathways; ko00561//Glycerolipid metabolism) (Supplementary Table 7). For plants, chilling tolerance is closely related to the composition, structure, and metabolic processes of membrane lipids (Zhang et al., 2019), and plants can adjust membrane stability and fluidity by changing the unsaturation of fatty acids in membrane lipids, which is important for resisting cold stress (Karabudak et al., 2014). With the development of biotechnology, the genetic engineering of rice cold tolerance has considerably progressed. Several genes related to cold tolerance in fatty acid metabolism pathways have been cloned and transferred to plants for functional research (Nair et al., 2009; Cao et al., 2010); for rice, *qPSST6*, a major gene involved in the synthesis of long-chain fatty acids, was identified as a cold-tolerance gene at the booting stage of rice (Sun et al., 2018). However, whether Os09g0444200 regulates cold tolerance by participating in lipid metabolism requires further construction of transgenic rice for verification. Moreover, as a newly identified CTBB regulator, the signaling transduction system and the downstream pathways of Os09g0444200 are still unclear. The biological functions of Os09g0444200 and its associated genomic variations need to be further confirmed using gene editing and high-efficiency overexpression transformation systems.

## Data Availability Statement

The datasets presented in this study can be found in online repositories. The names of the repository/repositories and accession number(s) can be found below: NCBI (accession: PRJNA688381).

## Author Contributions

LY and DZ conceived and designed the research. LY, LL, and PL participated in data analysis. JW, HZ, CW, FY, JC, CW, and LL performed material development, sample preparation, and data analysis. LY wrote the manuscript. DZ corrected the manuscript. All authors read and approved the final manuscript.

## Conflict of Interest

The authors declare that the research was conducted in the absence of any commercial or financial relationships that could be construed as a potential conflict of interest.

## Footnotes

1 http://www.appliedbiosystems.com

2 http://www.isbreeding.net

3 http://rice.plantbiology.msu.edu/

## Supplementary Material

Supplementary Figure 1 Clustering map of GO annotation of genes in 483.87 kb regions. The abscissa is the content of GO categories, and the left of the ordinate is the number of genes. This figure shows the gene classification of GO secondary functions in the context of all genes in the associated region.

Supplementary Figure 2 Clustering map of KEGG annotation genes in 483.87 kb regions. The ordinate is the name of the KEGG pathway, and the abscissa is the number of genes annotated under the pathway.

Supplementary Figure 3 Expression levels of the six candidate genes in DN430 and DF104 under normal condition measured by qRT-PCR. The results were statistically analyzed using Student’s *t*-test (^∗∗^*P* < 0.01).
