# Supplementary figures and images for: Identification of Candidate Genes Conferring Cold Tolerance to Rice (Oryza sativa L.) at the Bud-Bursting Stage Using Bulk Segregant Analysis Sequencing and Linkage Mapping
Source: Front Plant Sci. 2021 Mar 11;12:647239. doi: 10.3389/fpls.2021.647239 (PMC8006307; doi:10.3389/fpls.2021.647239)

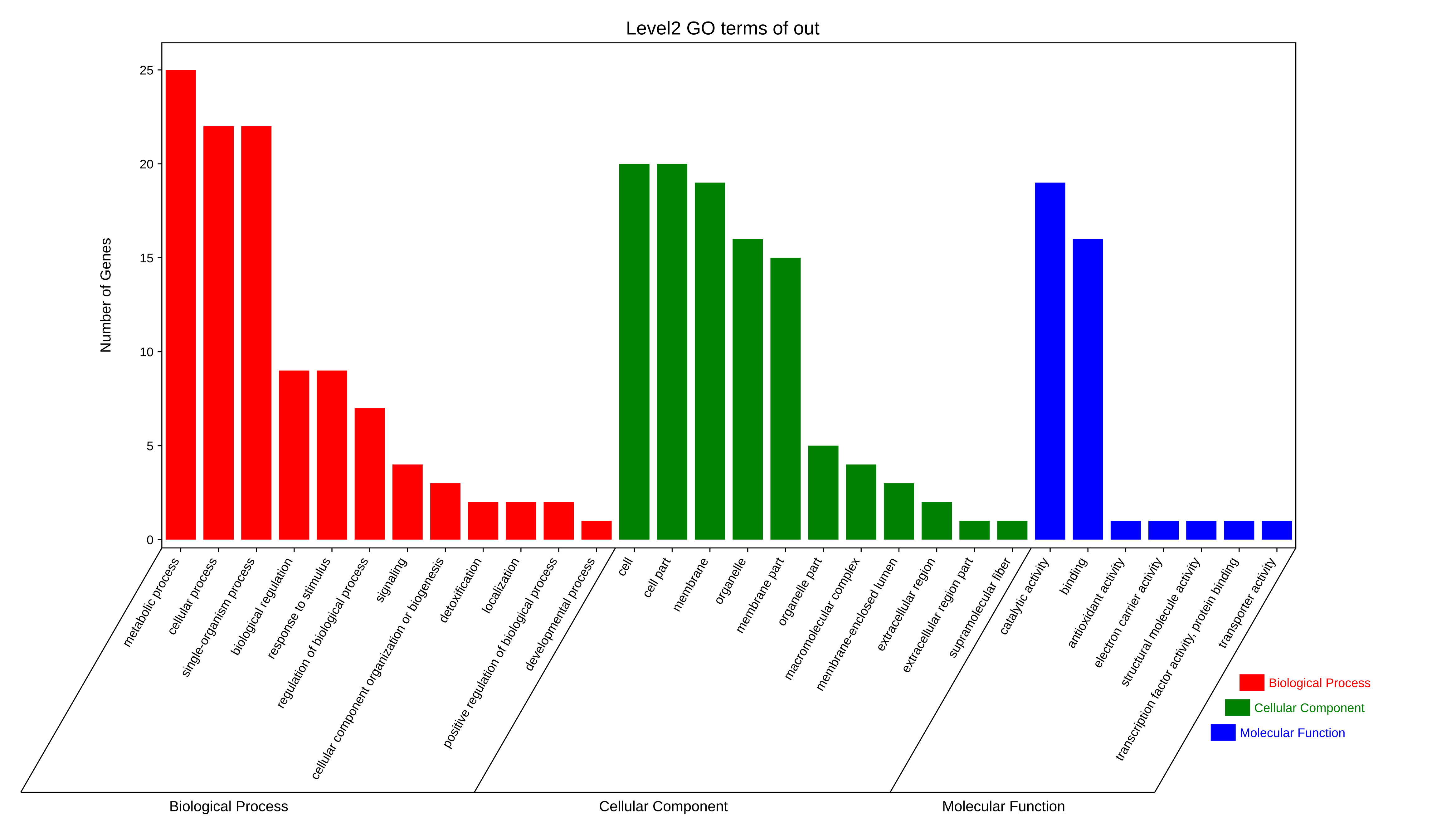

Supplement: Supplementary Figure 1 — Clustering map of GO annotation of genes in 483.87 kb regions. The abscissa is the content of GO categories, and the left of the ordinate is the number of genes. This figure shows the gene classification of GO secondary functions in the context of all genes in the associated region. [file Image_1.PNG]

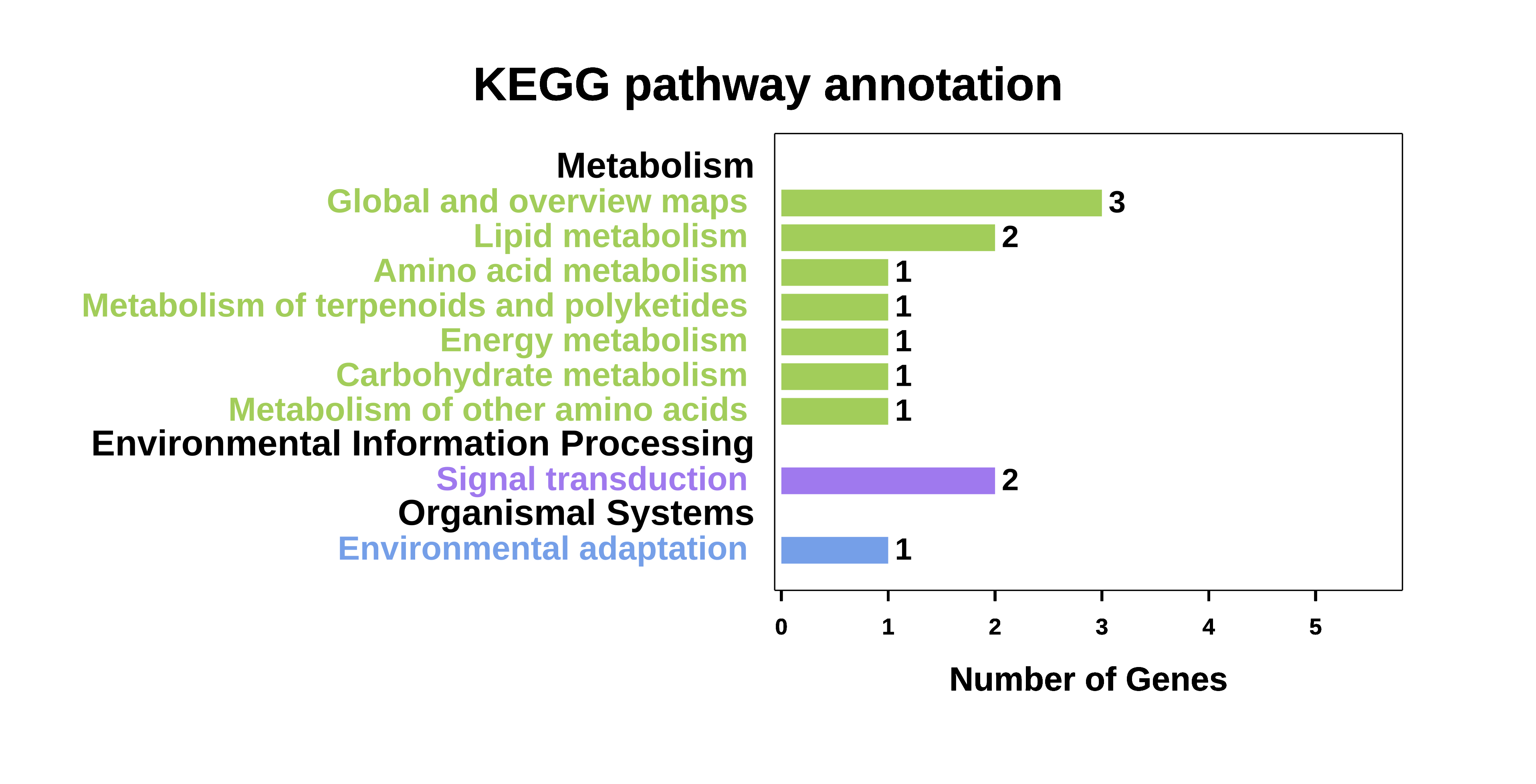

Supplement: Supplementary Figure 2 — Clustering map of KEGG annotation genes in 483.87 kb regions. The ordinate is the name of the KEGG pathway, and the abscissa is the number of genes annotated under the pathway. [file Image_2.PNG]

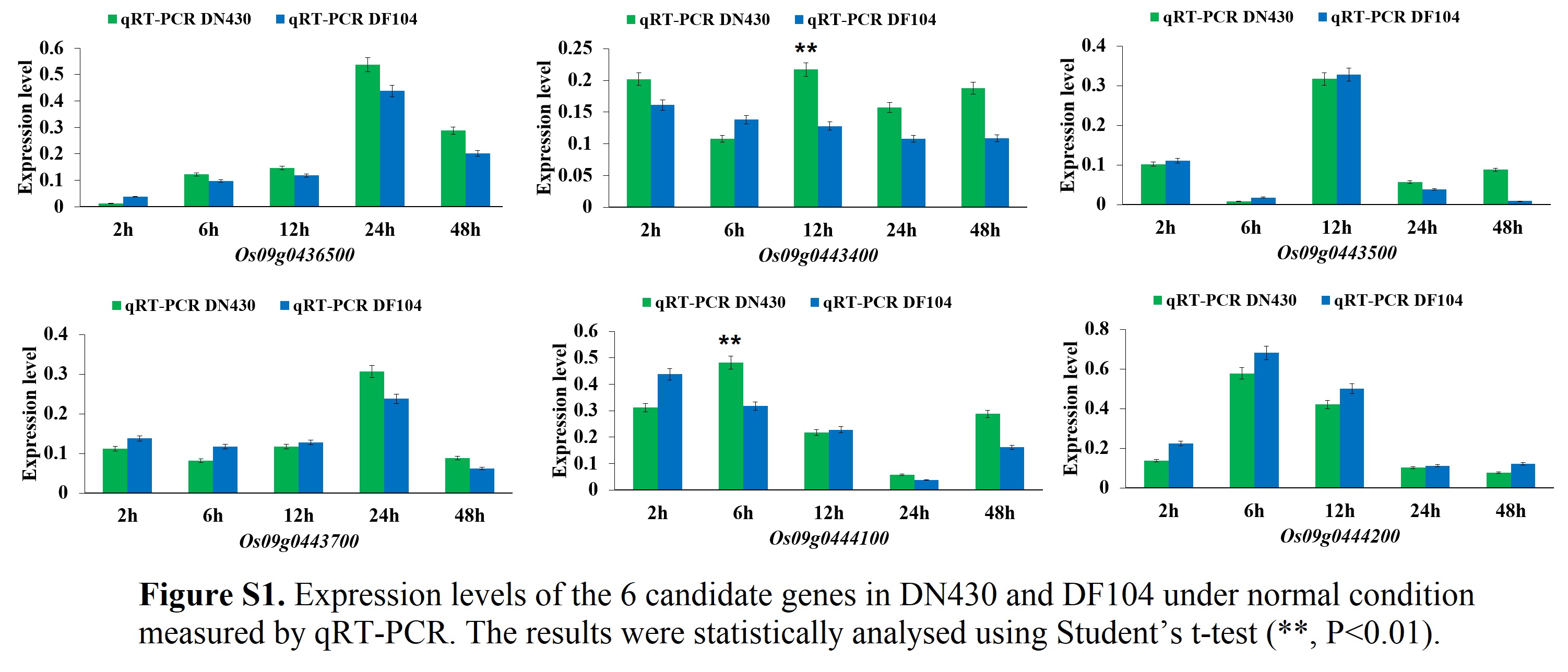

Supplement: Supplementary Figure 3 — Expression levels of the six candidate genes in DN430 and DF104 under normal condition measured by qRT-PCR. The results were statistically analyzed using Student’s t-test (∗∗P < 0.01). [file Image_3.JPEG]
